# Biocompatible micro-sized cell culture chamber for the detection of nanoparticle-induced IL8 promoter activity on a small cell population

**DOI:** 10.1186/1556-276X-6-505

**Published:** 2011-08-23

**Authors:** Yvonne Kohl, Gertie J Oostingh, Adam Sossalla, Albert Duschl, Hagen von Briesen, Hagen Thielecke

**Affiliations:** 1 Department of Cell Biology and Applied Virology, Fraunhofer Institute for Biomedical Engineering, 66386 St. Ingbert, Germany; 2 Department of Molecular Biology, University of Salzburg, 5020 Salzburg, Austria; 3 Department of Medical Engineering and Neuroprosthetics, Fraunhofer Institute for Biomedical Engineering, 66386 St. Ingbert, Germany; 4 Vanguard AG, 12623 Berlin, Germany

**Keywords:** micro-sized cell culture chamber, inflammation, nanoparticles

## Abstract

In most conventional *in vitro *toxicological assays, the response of a complete cell population is averaged, and therefore, single-cell responses are not detectable. Such averaging might result in misinterpretations when only individual cells within a population respond to a certain stimulus. Therefore, there is a need for non-invasive *in vitro *systems to verify the toxicity of nanoscale materials. In the present study, a micro-sized cell culture chamber with a silicon nitride membrane (0.16 mm^2^) was produced for cell cultivation and the detection of specific cell responses. The biocompatibility of the microcavity chip (MCC) was verified by studying adipogenic and neuronal differentiation. Thereafter, the suitability of the MCC to study the effects of nanoparticles on a small cell population was determined by using a green fluorescence protein-based reporter cell line. Interleukin-8 promoter (pIL8) induction, a marker of an inflammatory response, was used to monitor immune activation. The validation of the MCC-based method was performed using well-characterized gold and silver nanoparticles. The sensitivity of the new method was verified comparing the quantified pIL8 activation via MCC-based and standard techniques. The results proved the biocompatibility and the sensitivity of the microculture chamber, as well as a high optical quality due to the properties of Si_3_N_4_. The MCC-based method is suited for threshold- and time-dependent analysis of nanoparticle-induced IL8 promoter activity. This novel system can give dynamic information at the level of adherent single cells of a small cell population and presents a new non-invasive *in vitro *test method to assess the toxicity of nanomaterials and other compounds.

**PACS: **85.35.Be, 81.16.Nd, 87.18.Mp

## Background

There is a growing interest in improved test methods to assess biological effects of nanoparticles. Studies of cellular processes and determination of toxic effects of nanomaterials on cells are commonly based on examining the response of a cellular population, such as a cell monolayer, tissue, or organ [1-6]. In many biological assays, such as colorimetric, fluorometric, or chemiluminescent assays, the data are a result of the mean response of the complete cell population. In those assays, the signal of a single cell is lost in the signal caused by the large cell sample. A detectable signal, above the background noise, can be due to the response of a specific subset of cells within the population or by a response of the complete cell population. Especially when performing biological studies with nanoparticles, there might be a large variation in the response of the individual cells based on whether or not they came in contact with nanoparticles and, in addition, on the level of exposure, which is known to play an important role. Since an altered response in a low number of cells can be the trigger for certain diseases, such as autoimmunity, cancer, and neuronal diseases, the analysis of nanoparticle-induced responses of individual cells is of main importance [7,8]. Therefore, cell-based assays that can detect the response of a low number of individual cells are required. In addition, *in vitro *studies demonstrated differences in the behavior of cells isolated or in a cell population [9-11], showing that isolated single cells react in a different physiological manner compared to cells within a monolayer or cell suspension. New methodologies have to be established to bridge the gap between population and quantitative single-cell analysis. Technologies for the characterization of single cells, such as capillary electrophoresis (2D, 3D), polymerase chain reaction (PCR), single-cell gel electrophoresis, and elastography, are already used, but these are invasive and often time-consuming techniques [12-22]. Invasive techniques destroy the cell and consequently do not permit the detection of single living cells or to perform kinetics on one and the same cell. Flow cytometry is used to investigate nanoparticle-induced effects at the single-cell level but is not suitable for the characterization of adherent cells since the cells need to be in suspension. Detachment of the cells from the surface of the cell culture dish might alter their characteristics [23]. With regard to the application of single-cell analysis as pharmaceutical *in vitro *screening method, the goal of this study is the evaluation and validation of a non-invasive technique to characterize cellular processes of adherent biological cells on an individual level in a small defined cell population. Biological microelectromechanical systems (Bio-MEMS) present a suitable approach for analyzing a small amount of cells on a defined cell culture area. Recently, classical detection technologies like optical and electrochemical analysis and mass spectroscopy have been combined with the chip technology [24-26]. Dynamic single-cell culture arrays of isolated cells have enabled to determine the level of produced or secreted proteins but do not simulate the physiological conditions of a 2D cell culture [21,27]. Silicon nitride (Si_3_N_4_) has been used as matrix for cell-based assays due to its chemical, optical, and mechanical properties [28]. Only few studies exist on the biocompatibility of Bio-MEM-materials [29-33]. Currently, no Bio-MEMS exist for long-term culturing, and long-term observation of cell response features larger, more comparable cell culture area dimensions compared to the micro-sized cell culture chamber presented in this paper [32,34-40]. At current, no Bio-MEMS exist for long-term cultivation and non-invasive quantification of specific cellular responses of adherent individual cells in a small defined cell layer cultured on miniaturized Si_3_N_4 _membranes with cell culture areas smaller than 0.2 mm^2^. The use of a micro-sized chip-based cell culture system in combination with reporter cells presents a powerful tool for the analysis of small cell populations and will improve the evaluation of non-invasive *in vitro *test methods to observe sub-toxic effects on individual adherent cells in a small cell population under physiological conditions. This article introduces a miniaturized microcavity chip (MCC)-based method for the non-invasive analysis of nanoparticle-induced effects of adherent single cells in a small defined cell layer.

## Results and discussion

### Fabrication of the miniaturized microcavity chip

**Figure 1:**
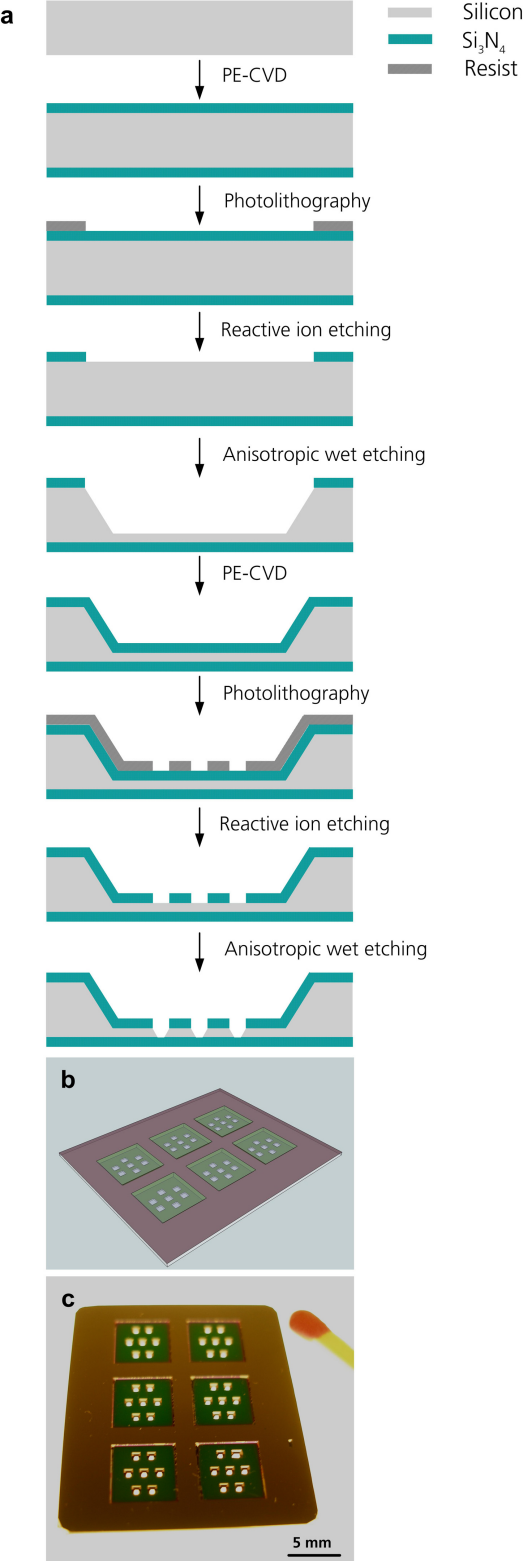
**The miniaturized cell culture chamber**. (**a**) Work flow of the fabrication. (**b**) Design of the MCC. The MCC contains 6 × 7 miniaturized cell culture chambers. (**c**) Photographic image of the microcavity chip. Scale bar 5mm.

An MCC was fabricated by semiconductor process technology (Figure 1a). The design focused on the improvement of the high-quality optical analysis of cellular reactions of a small cell population compared to conventional cell culture chambers. An 800-nm-thick transparent Si_3_N_4_ membrane forms the cell culture area with a surface of 0.16 mm^2^. Due to the positive optical and mechanical properties of Si_3_N_4_, the micro-sized culture chamber has optimal optical properties when using microscopic analysis methods. The seven individual miniaturized cell culture chambers in each cultivation segment guarantee a statistical analysis of the generated data. The MCC represents an array of miniaturized cell culture chambers for permanent non-invasive characterization of individual cells in a cell layer. The miniaturization of the cell culture area guarantees the observation of the complete cell culture area (Figure 1b, c).

Currently, the 800-nm-thick transparent Si_3_N_4 _membrane used in this study is the thinnest membrane layer available so far with good optical properties, allowing easy analyzing of individual cells in a cell culture layer with high optical quality. The six individual culture segments provide the opportunity to analyze different materials or concentrations under identical physiological conditions (Figure 1c).

**Figure 2:**
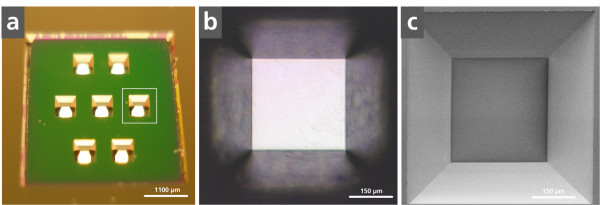
**Microscopic images of the miniaturized cell culture chamber with a Si_3_N_4 _membrane**. (**a**) Photographic image. Scale bar 1,100 μm. (**b**) Phase contrast microscopic image. Scale bar 150 μm. (**c**) Scanning electron microscopic image. Scale bar 150 μm.

Each of the six culture segments possesses seven individual microcavities which are used as cell culture chambers (Figure 2a). The addition of a test substance in one of the six culture segments guarantees a statistical analysis by the seven separate micro-sized cell culture chambers. The size of the Si_3_N_4 _membranes of the cell culture area (400 × 400 μm) (Figure 2b, c) was chosen to observe the whole area with one microscopic image (888 × 666 μm) and to guarantee a more physiologically realistic condition compared to single-cell analysis, since about 200 to 250 cells are present in each cavity and thus a small monolayer can be formed. To observe all cells of a cell layer in conventional cell culture chambers, the whole area has to be scanned, which is a very time-consuming procedure. The advantage of the miniaturized cell culture chamber is that the entire cell culture area can be analyzed quickly with better optical quality and without any changes of the cell behavior.

Another advantage of the MCC is that optimal focusing is possible, whereas polystyrene membranes of conventional cell culture dishes only allow focusing in the center of the cell culture area due to edge effects. The Si_3_N_4_-cell culture area of the miniaturized system possesses a square shape due to its production process. Due to the etch process, the end walls are positioned in an angle of 54.7° amplifying the optical properties of the cavity membrane due to the reduced edge effects. Preliminary experiments showed that the round shape of conventional cell culture chambers, like 96-well microplates or 384-well microplates, resulted in edge effects, leading to unfocused microscopic images of the cells. Additionally, the correlations between fluorescent and bright-field images did not conform to each other when using conventional polystyrene cell culture chambers. In contrast, the developed micro-sized cell culture chamber reduced the working distance during microscopy due to the 800-nm-thin Si_3_N_4 _membrane. In addition, due to the square shape of the cell culture chambers, the edge effects are minimized resulting in clear focused microscopic images with analogy bright-field and fluorescent images with high optical quality. Furthermore, Si_3_N_4 _features minimal auto-fluorescence in comparison to polystyrene.

Currently, only few microsystems exist for non-invasive analysis of specific reactions of individual cells in a small adherent cell population via optical methods [32,38]. Stangegaard et al. described a polymethylmethacrylate (PMMA) chip as micro cell culture system with a cell culture area of 99 mm^2 ^[32]. In comparison to the PMMA-micro cell culture system, the established MCC with its 800-nm-thin Si_3_N_4 _membranes offers a better optical quality and can also be used for scanning electron microscopy (SEM). Compared to the conventional fluorescence-based analysis techniques, the combination of a reporter cell line and the MCC presents a more sensitive and cost-efficient *in vitro *method. Advantages of the quantitative analysis via MCC are the low sample volume, the small amount of test materials, the capture of the complete cell culture area with high optical quality, and thus the possibility to statistically analyze the variations between the individual cell responses.

### Analysis of the biocompatibility of the MCC

**Figure 3:**
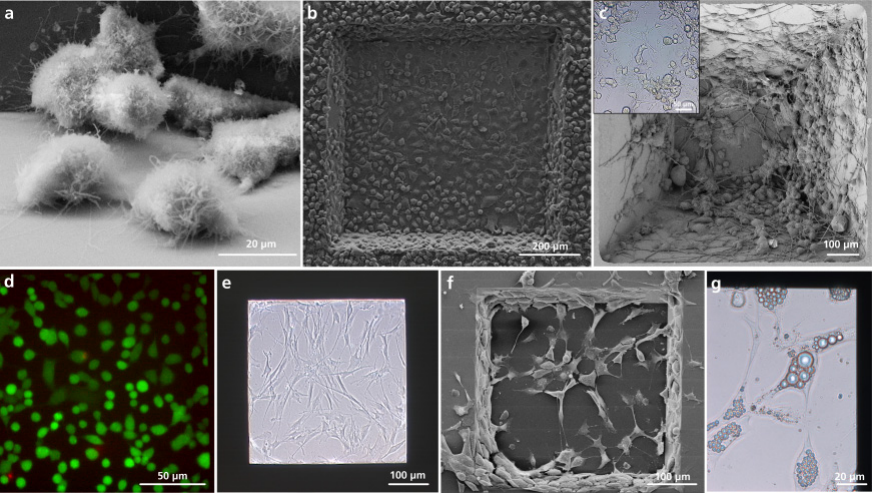
**Microscopic images of different cell types cultured in the miniaturized cell culture chamber**. (**a**) Scanning electron microscopic image of A549 cells on the Si-sidewalls. Scale bar 20 μm. (**b**) Scanning electron microscopic image of A549 cells after 7 days of culture on the Si_3_N_4 _membrane. Scale bar 200 μm. (**c**) Scanning electron microscopic image of PC-12 cells 8 days after neuronal differentiation. Scale bar 100 μm. Small box: bright-field image of neuronal differentiated PC-12 cells. Scale bar 50 μm. (**d**) Fluorescence microscopic image of A549 cells after 7 days of cultivation after FDA/PI staining. Scale bar 50 μm. (**e**) Bright-field microscopic image of proliferating hMSCs after 7 days. Scale bar 100 μm. (**f**) Scanning electron microscopic image of hMSCs after 18 days adipogenic differentiation. Scale bar 100 μm. (**g**) Bright-field image of adipogenic differentiated hMSCs. Scale bar 20 μm.

The biocompatibility of the evaluated microcavity chip was analyzed by culturing human bronchial epithelial cells (A549 cells) in the miniaturized cell culture chamber for 48 h (Figure 3). The cells adhered onto the Si_3_N_4 _membranes and showed characteristic morphologies. Scanning electron microscopic images after 7 days of cultivation of A549 cells confirmed their adherence to the Si_3_N_4 _membrane (Figure 3b). Moreover, the cells did not only adhere to the Si_3_N_4 _membrane but also to the Si sides (Figure 3a, b). The viability of the A549 cells was verified after 7 days of proliferation via fluorescein diacetate (FDA)/propidium iodide (PI) staining (Figure 3d). The viability after this prolonged incubation period was 96.2 ± 0.3%. Furthermore, the suitability of the miniaturized cell culture chambers for cultivation and differentiation of sensitive *in vitro *systems was determined.

As sensitive *in vitro *system, PC-12 cells (rat adrenal pheochromocytoma cells) were grown in the microcavity. These cells are used as model cells in tissue engineering [41,42]. After adding the differentiation stimulus nerve growth factor to the cell culture medium, the suspension cells started to adhere and form neuronal networks (Figure 3c). Mesenchymal stem cells (MSCs) were used as a model for a sensitive *in vitro *system [43]. The morphology of the human MSCs (hMSCs) during proliferation is comparable to the morphology of the cells cultured on polystyrene membranes as it is common in conventional cell culture chambers like 96-well microplates (Figure 3e). The adipogenesis was used to determine the effect of miniaturization on the differentiation capacity of hMSCs. Human MSCs were cultured for 18 days in adipogenic differentiation medium. Lipid droplets, which were formed as a result of adipocytes, are visible by bright-field microscopy (Figure 3g). Scanning electron microscopy (SEM) images of the adipogenic differentiated hMSCs show a clear increase of adipogenic differentiated hMSCs, also in the corner areas of the microcavity (Figure 3f). The performed studies verify the biocompatibility of the Si_3_N_4 _membrane and the suitability of the microcavity for *in vitro *studies. A549 cells as well as hMSCs proliferate in the microcavity. Furthermore, we are the first to demonstrate the possibility to induce adipogenic differentiation of hMSCs as well as a neuronal differentiation of PC-12 cell in the microcavity with a cell growth area of 0.16 mm^2^. Due to the high need for MSCs in the field of tissue engineering, the micro-sized cell culture area opens new potential for culturing and differentiation of 3D MSC cultures as well as studying stem cell niches using relatively low numbers of cells which also allows the inclusion of more repetitions and treatments. Such studies could provide insight in cancer stem cell research, since miniaturization allows a detailed observation of the complete cell population in the cell culture chamber. The microchip combined with neuronal cells provides a basis for new methods for research on neuronal diseases like Alzheimer or Parkinson disease, for the development of new sensitive drug screening methods and for the quantification of toxicodynamic and toxicokinetic effects.

### Application of the MCC for the analysis of nanoparticle-induced effects

After confirmation of the biocompatibility of the evaluated miniaturized cell culture chambers, the system was validated for the non-invasive quantification of IL8 gene promoter activations of individual cells of a small cell population. Currently, much research is ongoing to determine potential effects of nanoparticles on health of workers and consumers. The amounts of engineered nanoparticles with a range of different sizes and shapes and made from different materials are steadily growing, and there is a need to determine the biological response to these novel materials. In this respect, the immune system is of special interest, since one of the main functions of the immune system is to deal with foreign materials [44].

In order to determine whether or not the MCC method could be suitable for the analysis of nanoparticle-induced immunomodulatory effects, a stable transfected A549 reporter cell line, containing the IL8 promoter sequence linked to the gene for green fluorescence protein (pIL8-GFP), was established. The sequence of the IL8 promoter was placed before the GFP sequence, whereby GFP was used as a reporter gene. IL8 promoter activation resulted in the generation of GFP which was accumulated within the cell. Since the original IL8 gene has not been replaced, the analysis of IL8 expression by conventional methods is still feasible. Beyond that, the combination of the miniaturized cell culture chamber and the transfected reporter cell line pIL8-GFP A549 allows the detection of specific IL8 promoter activity of individual cells in a small adherent cell population. First of all, the cells were stimulated by a pro-inflammatory stimulus to determine whether the cells respond in an appropriate manner. Recombinant human tumor necrosis factor alpha (rhTNF-alpha), a cytokine involved in local and systemic inflammations, was added to the cell culture. The GFP expression of the transfected pIL8-GFP A549 cells verifies an IL8-coupled inflammatory response. The kinetics and stability of GFP was determined by stimulating the A549 cells with the rhTNF-alpha. Stimulation with rhTNF-alpha showed a dose-dependent increase in GFP production which peaked when using 20 ng/ml TNF-alpha (unpublished observation). Moreover, the cell line could be kept in culture for more than 1 month without a loss of responsiveness to general cellular stimuli.

**Figure 4:**
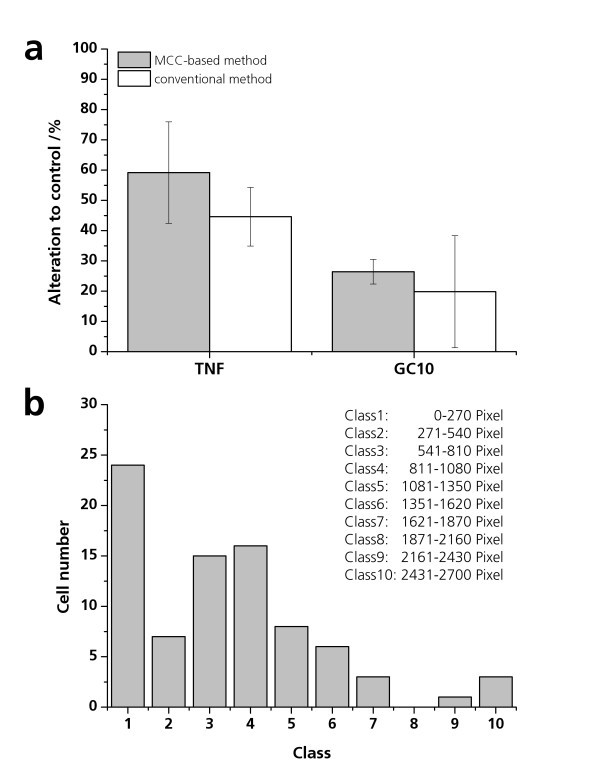
**GFP expression of TNF-alpha- and GC10-exposed pIL8-GFP A549 cells**. pIL8-GFP A549 cells were cultured in the microcavities and exposed to 20 ng/ml TNF-alpha or 30 μg/ml GC10 for 24 h under physiological conditions. In parallel, 10,000 pIL8-GFP A549 cells were seeded in 96-well microplates and stimulated with 20 ng/ml TNF-alpha and 30 μg/ml GC10 for 24 h. After the exposure time, the GFP expression of the pIL8-GFP A549 cells in the microcavities was analyzed by fluorescence microscopy. The percentage of GFP-expressing cells in the microcavity was calculated via the software analysis. The GFP expression of the pIL8-GFP A549 cells in the 96-well micro plate was quantified by fluorescence spectrometry. The percentage of GFP expression is pictured as alteration to the untreated control (alteration to control/percent). (**b**) pIL8-GFP A549 cells were treated for 24 h with 20 ng/ml TNF-alpha in the microcavities. The GFP expression of 90 individual cells was quantified. The classes of the fluorescence intensities (*x*-axis: class of GFP intensity) and its frequency (*y*-axis: frequency) is presented.

After 24 h exposure of the pIL8-GFP A549 cells with 20 ng/ml TNF-alpha, the GFP expression was quantified via fluorescence spectrometry using a 96-well microplate and via fluorescence microscopy using the micro-sized cell culture chamber. The comparison of the two different methods results in a higher response when using the MCC-based technique (Figure 4a). By the miniaturized method, GFP expression was detectable in 59.2 ± 16.8% of the cells in the microcavity compared to the untreated control (Figure 4a). Via a 96-well microplate, an increase of fluorescence intensity of 44.6 ± 9.7% was proven (Figure 4a). Thereafter, the fluorescence intensity of 90 individual GFP-expressing pIL8-GFP A549 cells was quantified after incubation with TNF-alpha (20 ng/ml) in the micro-sized cell culture chamber. The fluorescence intensity of each individual cell was quantified digitally as pixel number. The pixel number of the 90 analyzed cells varied between 0 and 2,700 pixels per cell. The histogram of the fluorescence intensity evidenced that most stimulated cells have fluorescence intensities with values less than 270 pixels (Figure 4b). This result revealed that the MCC-based system is very sensitive and feasible for quantifying GFP expression and distinguishing the fluorescence intensity of individual cells in a small cell population.

Chemicals but especially particles can interact with single cells within a cell population and only induce a response at a certain threshold concentration, which varies from cell to cell, e. g., depending on the cell cycle stage or on previous exposures. Therefore, the analysis and quantification of single-cell responses will provide important information on the toxicity of the tested materials. The MCC-based method is therefore qualified as new non-invasive *in vitro *method for analyzing single-cell responses of adherent cells under physiological conditions.

In order to detect the suitability to use the developed method for nanotoxicology studies, two nano-sized materials (gold nanoparticles (GC10) and silver nanoparticles (SC10)) have been used for validating the new non-invasive method. Before investigating the effect of the nanoparticles on the IL8 promoter activation, they were characterized physicochemically (Table 1). The detected zeta-potential is a characteristic for uncoated nano-scaled gold and correlates to the data described in the literature [45-47].

**Table 1:** Physicochemical characterization of the used nanoparticles

Nanoparticle	Material	Diameter (nm)	zeta-**potential (mV)**	Absorption maxima (nm)
GC10	Gold	9.0 ± 0.03	-33.8 ± 1.82	515
SC10	Silver	7.37 ± 0.03	-41.03 ± 0.9	395
MC100	Magnetite	63.8 ± 0.37	-4.5 ± 0.55	320

**Figure 5:**
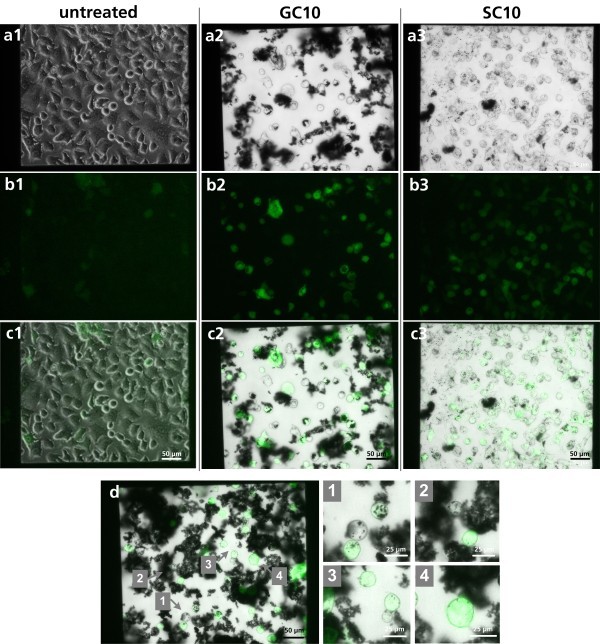
**Microscopic images of SC10- and GC10-treated pIL8-GFP A549 cells in the microcavity**. pIL8-GFP A549 cells were treated for 48 h with (2) GC10 and (3) SC10 under physiological conditions. (**a**) Bright-field image. Scale bar 50 μm. (**b**) Fluorescence image. Scale bar 50 μm. (**c**) Overlay of the bright-field and the fluorescence images. Scale bar 50 μm. (**d**) Overlay of the bright-field and the fluorescence images of individual GC10-exposed pIL8-GFP A549 cells. Sections of this image are pictured in (d1 to d4). Beside individual GFP-expressing pIL8-GFP A549 cells interacting with nanoparticle aggregates (d1, d2), also GFP-expressing cells with few or less nanoparticle interaction were observed (d3, d4).

To determine the inflammatory effect of GC10, pIL8-GFP A549 cells were cultured in presence of 30 μg/ml nanoparticle suspension in the microcavities (0.16 mm^2^) and in the well of a 96-well plate (34 mm^2^) for 24 h. For every cavity, the total number of cells and the number of fluorescent cells were determined by microscopy, and the ratio of fluorescent cells was calculated. The quantification of the fluorescence and bright-field images resulted in an increased amount of fluorescent cells (26.44 ± 4.09%) in comparison to the conventional method (19.8 ± 18.5%) (Figure 4a). In addition, the fluorescence spectrometry resulted in a major standard deviation. In contrast, the MCC-based method shows a small standard deviation, which indicates that it is a very sensitive and reproducible system. For correlating the amount of nanoparticles and the inflammatory status of a single cell, pIL8-GFP A549 cells were incubated in 30 μg/ml GC10 or SC10 for 48 h. By fluorescence microscopy, it was observed that the nanoparticles were not located homogeneously on the cells and on the membrane (Figure 5). However, no correlation was observed between the amount of nanoparticles on the cells and the IL8 promoter activation. Nevertheless, the overlay of the bright-field image (Figure 5a) and the fluorescence image (Figure 5b, 1 and 2) of the GC10- and SC10-treated pIL8-GFP A549 cells in the microcavity allows quantification of the fluorescence intensity and thus the inflammatory status of single cells.

**Figure 6:**
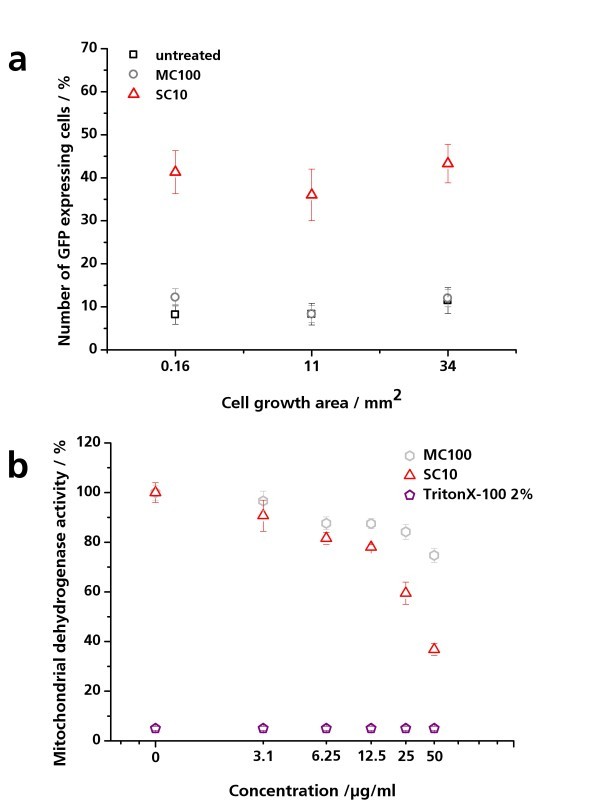
**Effect of nanoparticles on pIL8-GFP A549 cells**. (**a**) Effect of miniaturization on nanoparticle-induced inflammation in pIL8-GFP A549 cells. pIL8-GFP A549 cells were cultured on three different growth areas (0.16, 11, and 34 mm^2^) and exposed to 20 μM SC10 and 20 μM MC100 for 24 h under physiological conditions. The percentage of GFP-expressing cells per growth area was analyzed by fluorescence microscopy. The amount of GFP-expressing pIL8-GFP A549 cells in relation to the cell growth area is depicted. The results are presented as mean of three independent experiments ± SD. (**b**) Concentration-dependent effect of nanoparticles on mitochondrial dehydrogenase activity of pIL8-GFP A549 cells. pIL8-GFP A549 cells were exposed to 0 to 50 μg/ml SC10 und MC100 for 24 h under physiological conditions. Triton X-100 was used as positive control. Via WST-1 assay the mitochondrial dehydrogenase activity was quantified. Untreated cells were set as 100%. The results are presented as mean of three independent experiments ± SD compared to the untreated control.

The determination of the effect of miniaturization on nanoparticle-induced inflammatory cell responses resulted in a basal amount of untreated pIL8-GFP A549 cells varying between 8% and 11%, for all tested cell culture areas (Figure 6a). This is in agreement with previous experiences that A549 undergoes some degree of activation by normal cell culture procedures and that IL8 induction is a particularly sensitive signal. After MC100 exposure, the amount of GFP-expressing cells increased slightly but was still at the level of the untreated control. After SC10 exposure, the amount of fluorescent cells increased to 41.3 ± 5.1% (0.16 mm^2^), 36.0 ± 6.2% (11 mm^2^), and 43.3 ± 4.5% (34 mm^2^) (Figure 6a). The results obtained showed that the growth area had no influence on the cell response.

**Figure 7:**
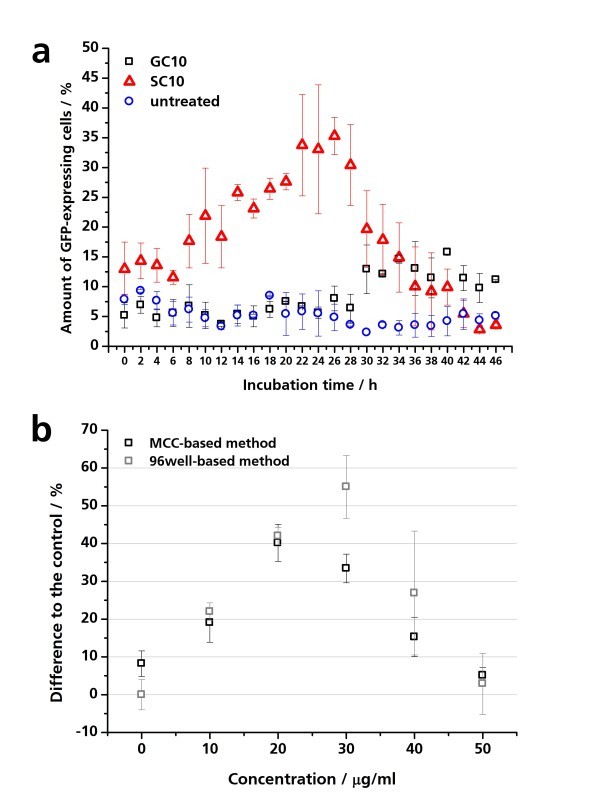
**Concentration- and time-dependent effects of nanoparticles on the GFP expression of pIL8-GFP A549 cells**. (**a**) pIL8-GFP A549 cells were cultured in the microcavities and exposed to 30 μg/ml GC10 and SC10 for 48 h. The GFP expression of the pIL8-GFP A549 cells was analyzed via fluorescence time-lapse microscopy. The percentage of GFP-expressing cells was quantified using the software analysis. (**b**) pIL8-GFP A549 cells were treated with 0 to 50 μg/ml SC10 for 24 h in the microcavity under physiological conditions. Parallel 10,000 pIL8-GFP A549 cells were cultured and treated in a 96-well microplate with 0 to 50 μg/ml SC10. The GFP expression of the pIL8-GFP A549 cells in the microcavities was analyzed by fluorescence microscopy and the GFP expression of the cells in the microplate by fluorescence spectrometry.

The cytotoxic effect of SC10 and MC100 was evaluated using the WST-1 assay. MC100 induced a concentration-dependent cytotoxicity but a low decrease in mitochondrial activity with a maximum reduction of 20% when cells were treated with 50 μg/ml MC100. In contrast, SC10 had an IC_50 _value of 27 μg/ml in pIL8-GFP A549 cells, which correlated with the effect of SC10 on IL8 promoter activation (Figure 6b and 7b). The reduction in fluorescence intensity at higher concentrations could therefore be caused by the cytotoxic effects of SC10. A maximal reduction of the mitochondrial activity of 38% was found when cells were treated with 50 μg/ml SC10 (Figure 6b).

Besides the threshold-dependent detection of inflammatory reactions, the usability of the MCC-based system to determine time-dependent inflammatory processes was tested. By time-lapse microscopy, SC10 induced a time-dependent increase of the amount of GFP-expressing pIL8-GFP A549 cells in the microcavity. After 26 h, the percentage of fluorescent cells decreased to the fluorescence level of untreated pIL8-GFP A549 cells (Figure 7a). The amount of GFP-expressing GC10-treated cells remained on the control level, and after 30 h, the amount of fluorescent cells increased to 12.9 ± 4.1% and ranges in the following 18 h between 9.8 ± 2.5% and 15.8 ± 0.5% (Figure 7a).

To test the use of the micro-sized cell culture for determination of threshold-dependent effects, pIL8-GFP A549 cells were incubated with 0 to 50 μM SC10 for 24 h at 37°C. Ten micrograms per milliliter of SC10 induced an increase in fluorescence intensity of 25%, and 20 μg/ml induced a significant increase of 40% (Figure 7b), whereas concentrations higher than 40 μg/ml caused no significant increase in fluorescence intensity compared to the untreated control. Inflammatory effects as well as cytotoxic effects are threshold-dependent effects. Low concentrations leading to an inflammatory process could cause cytotoxic effects, but normally this is not the case. If a cytotoxic effect is induced, the concentration is often too high to activate the inflammation-specific pathways in the cells. In our experiments, the exposure time of 24 h concentrations up to 20 μg/ml resulted in a significant IL8 promoter activation quantified as GFP expression and concentrations higher than 30 μg/ml resulted in a significant decrease of cell viability as analyzed by WST-1 assay resulting in less GFP expression (Figure 6b and 7b).

The combination of the miniaturized cell culture chamber and the transfected reporter cell line pIL8-GFP A549 realizes the establishment of a chip-based *in vitro *method as non-invasive technique for detecting inflammatory processes of adherent cells in a small cell population. Compared to the 96-well microplates, the new miniaturized cell culture chamber enables a fast and sensitive quantification of IL8 promoter activations that is based on the analysis of individual cells within a population.

It has been described that the physical properties of nanoscale materials can interfere with the analysis of toxicological parameters [48,49]. The MCC-based method is based on optical analysis followed by digital quantification of the induced GFP expression of every individual cell in the microcavity. One advantage of the miniaturized method is the recording of the complete cell culture area in one image. Hence, every individual cell response is involved in the assessment of the inflammatory status. By observing every individual cell, the interference of the physical properties of the nanoparticles with the fluorescence spectrometric analysis was avoided. Besides reproducibility and sensitivity, the use of the miniaturized system for the detection of threshold-dependent effects was tested. The comparison of the data obtained using a 96-well microplate and the developed micro-sized cell culture chamber verified the suitability of the microcavities as biocompatible cell culture chamber with better optical quality and the suitability of the MCC in combination with the transfected reporter cell line pIL8-GFP A549 as new non-invasive *in vitro *method for the continuous observation of GFP expression and the quantification of concentration- and time-dependent nanoparticle-induced IL8 promoter activation in adherent cells of a small cell population.

## Conclusions

The goal of this study was to establish a biocompatible micro-sized cell culture chamber and to prove its applicability to determine nanoparticle-induced effects of an individual cell of a small cell population.

The need to develop non-invasive *in vitro *methods to detect nanoparticle-induced effects of a small cell population is high and can be illustrated in conjunction with the European chemical regulation REACH [50]. Previous methods for nanotoxicity studies are OECD standardized techniques; most of these ignore the individual differences within a cell population and can therefore lead to misinterpretations. The development of the MCC, described in this manuscript, allowed us to culture cells in a way in which their behavior is comparable to that observed in conventional cell culture systems. Compared to macro-scale cell culture chambers, the MCC offers the opportunity for culturing, long-term observation, and manipulation of a small amount of cells on a defined cell culture area. Using this non-invasive system, individual cells could easily be observed and specific cell reactions could be quantified.

The bottom of the miniaturized cell culture chambers was made of Si_3_N_4 _membranes to ensure biocompatibility as well as excellent optical properties for cell analysis. The small size of cavities enables a high number of cavities on each chip and facilitates the performance of many independent assays on one plate. The funnel-shaped cavities avoid the appearance of meniscuses, and therefore, the total cell growth area can be used for analysis. Moreover, the miniaturization allows the microscopic analysis of the entire cell population in the cavity in one microscopic field. The detection of the complete cell layer guarantees reproducible results without any subjective choice of representative areas of the cell monolayer.

Besides the advantage of convenient handling, the microscopic analysis of small amounts of cells and nanomaterials increase the through-put rate of the experiments, resulting in a time- and cost-effective method. The established cell culture chamber (0.16 mm^2^) is a biocompatible chamber with the thinnest (800 nm) transparent cell culture layer existing, resulting in high optical quality. Therefore, the proposed *in vitro *method bridged the gap between population measurements and quantitative single-cell analysis. Such a non-invasive system could be used to investigate the nano(immuno-)toxicity on an individual cell level, followed by selective quantitative analysis of the induced intensity.

## Methods

### Fabrication of the miniaturized cell culture chamber

A miniaturized microcavity chip (MCC) (length 3 cm, width 2.5 cm) was fabricated by semiconductor process technology. Base material was a 500- μm-thick < 100 > orientated silicon (Si) wafer, coated double-sided with an 800-nm-thick silicon nitride (Si_3_N_4_) layer. Design and fabrication are shown in Figure 2. The MCC consist of six cavities (length of the outline 4,000 × 4,000 μm, depth 400 μm), where each have seven separate funnel-shaped microcavities (length of the outline 400 × 400 μm, depth 100 μm). These represent the miniaturized cell culture chambers with a Si_3_N_4 _membrane and a growth area of 0.16 mm^2^. Prior to the application of the MCC for biological analysis, they were autoclaved at 121°C, 2 bar, for 15 min.

### Cell lines and culture conditions

All cell culture reagents were obtained from Invitrogen (Karlsruhe, Germany), unless stated otherwise. The human lung epithelial carcinoma cells A549 (ATCC no. 107) were cultured in RPMI medium supplemented with L-glutamine (4 mM), penicillin (100 U/ml), streptomycin (100 μg/ml), and 10% (*v*/*v*) fetal calf serum (FCS). PC-12 cells (rat adrenal pheochromocytoma cells, ATCC no. 159) were cultured in RPMI medium supplemented with L-glutamine (4 mM), penicillin (100 U/ml), streptomycin (100 μg/ml), 10% (*v*/*v*) horse serum, and 5% (*v*/*v*) FCS. For neuronal differentiation, RPMI medium was supplemented with 0.5% horse serum, 0.25% FCS, and 1% nerve growth factor. Human mesenchymal stem cells (hMSCs) were isolated from the bone marrow of human thighbone of human donors as described in literature [51]. The thighbones were kindly provided from the Protestant hospital in Zweibrücken (Germany) from Dr. M. Maue and Dr. Hassinger. Dr. E. Gorjup (Fraunhofer IBMT, St. Ingbert, Germany) isolated the hMSCs with a declaration of consent of each patient.

hMSCs were cultured in alpha-MEM supplemented with penicillin (50 U/ml), streptomycin (50 μg/ml), and 15% (*v*/*v*) heat-inactivated FCS (proliferation medium). For adipogenic differentiation, the proliferation medium was exchanged by differentiation medium (alpha-MEM supplemented with penicillin (50 U/ml), streptomycin (50 μg/ml) and 10% (*v*/v) FCS, 100 ng/ml insulin, 100 mM dexamethasone, 200 μM indomethacin, and 500 μM isobuthylmethylxanthine. Stable clones of pIL8 GFP-transfected A549 cells (A549 pIL8 GFP, see below) were cultured in the RPMI medium (supplemented with L-glutamine (4 mM), penicillin (100 U/ml), streptomycin (100 μg/ml), and 10% (*v*/*v*) FCS) in the presence of G418 (0.5 mg/ml final concentration).

Cells were maintained in a 5% CO2 humidified atmosphere at 37°C.

### Experimental procedure

**Figure 8:**
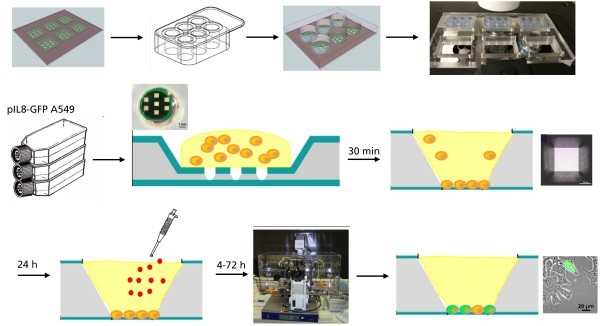
**Schematic experimental design**. To reduce the evaporation of the cell culture medium, a biocompatible cell culture chamber was positioned on the top of the MCC. Each chamber of the covered silicone FlexiPerm^® ^chamber includes seven individual miniaturized microcavities for statistical analysis of the experimental data. For each experiment, 100 μl cell suspension (100,000 cells/ml) were placed in each of the six culture segments. After 30 min, the cells adhered on the Si_3_N_4 _membrane. The segments of the cell culture chamber were filled with 100 μl cell culture medium. After 24 h of cell proliferation, the cells were exposed to the nanoparticles by aspirating the medium, washing the cells with PBS, and adding the nanoparticle-containing medium. After the exposure time, the cells were analyzed microscopically.

The experimental design is schematically depicted in Figure 8. To reduce the evaporation of the cell culture medium in the micro-sized cell culture chambers, a biocompatible cell culture chamber was positioned on the top of the MCC. Each chamber of the covered silicone FlexiPerm^® ^chamber (Greiner Bio-One, Frickenhausen, Germany) includes seven individual miniaturized microcavities for statistical analysis of the experimental data. For each experiment, 100 μl cell suspension (100,000 cells/ml) were placed in each of the six culture segments. After 30 min, the cells adhered onto the Si_3_N_4 _membrane. The segments of the cell culture chamber were filled with 100 μl cell culture medium. After 24 h of cell proliferation, the cells were exposed to the nanoparticles by aspirating the medium, washing the cells with PBS and adding the nanoparticle-containing medium. After the exposure time, the cells were analyzed microscopically. The total number of cells and the number of fluorescent cells were counted, and the percentage of GFP-expressing cells was calculated.

### Generation of the stably transfected reporter gene cell line

The host cells used for this study were A549 cells (ATCC no. 107). The human A549 cell line was transfected with an expression vector encoding green fluorescence protein (GFP) and an insert that encodes for the IL8 promoter region. The pTurboGFP-PRL expression vector was obtained from Evrogen (Moscow, Russia). This construct is a circular bacterial DNA which contains genes coding for ampicillin and neomycin resistance, allowing selection in respective bacteria and after transfection in human cells. Essential is that the construct also contains the GFP gene, as a reporter gene. The IL8 promoter sequence was amplified from human genomic DNA (Roche Diagnostics GmbH, Mannheim, Germany) by reversed transcriptase polymerase chain reactions (RT-PCR) using the forward primer 5'-ata ctc gag ggg tac ctt cgt cat act ccg tat ttg ata agg aac a-3' and the reverse primer 5'-aga att cgc ata gat ctt ccg gtg gtt tct tcc tgg ctc tt-3', containing the restriction enzyme sequences for Xho I and Eco RI, respectively, to allow cloning into the multiple cloning site. PCR with these primers resulted in an IL8 promoter fragment of 250 bp (NCBI NM 000584). The promoter fragment was chosen to include the main regulatory sites required for functional control of transcription. After cloning and plasmid isolation using standard techniques, A549 cells were transfected using Effectene (Qiagen, Hilden, Germany) following the distributors' instructions. After transfection, cells were cultured in the presence of 0.5 g/l G418 (gentamicin). The single-cell-derived clones of viable cells containing the insert, as verified by RT-PCR, were expanded. Batches of the stably transfected cell lines were frozen in liquid nitrogen, and individual aliquots of the stably transfected cells were not placed in culture for more than 1 month.

### Physicochemical characterization of the nanoparticles

To ensure a good comparability of our results with those obtained in other studies, we have chosen nanoparticles which have been selected by the National Institute of Standards and Technology as certified reference materials for preclinical biomedical research. The commercially available gold nanoparticle solutions, synthesized by the Frens method [52], were purchased from BBInternational (Cardiff, UK). These particles were spherical gold nanoparticles, 10 nm in size (type gold colloid GC10). The utilized silver nanoparticles (SC10) with a diameter of 10 nm were purchased from PlasmaChem GmbH (Berlin, Germany). The iron oxide nanoparticles (MC100) with a mean diameter of 100 nm possess a starch shell and are also commercially available (Chemicell GmbH, Berlin, Germany). The colloidal aqueous nanoparticle suspensions were sterile filtered to exclude any bacterial contamination (pore size 0.22 μm). The nanoparticles were characterized by dynamic light scattering, surface charge (zeta potential), and by absorption spectroscopy. For determining the size distribution of the nanoparticles, dynamic light scattering measurements have been performed on a Malvern Zeta Sizer Nano ZS (Malvern Instruments Ltd., Worcestershire, UK) using disposable clear zeta cells (DTS 1060C). The average diameter and polydispersity index (PDI) were provided by the instrument using general purpose analysis. The zeta average diameter and PDI reported herein were obtained as the average of three independent measurements (10 repetitions per measurement) performed on each sample. Zeta potential measurements were performed using a Malvern Instruments Zetasizer Nano (Malvern Instruments Ltd), operating with a variable-power (5 to 50 mW) He-Ne laser at 632 nm. Measurements were taken in zeta cells (DTS 1060C) at 25°C and repeated three times (10 repetitions per measurement) for each sample. UV-visible (UV/Vis) absorption spectra were taken on a two-beam UV/Vis spectrometer (Lambda 950, Perkin Elmer, WalthamMassachusetts, USA). The UV-visible absorption spectra of both gold nanoparticle suspensions were recorded at room temperature. For the experiments, the wavelength ranging from 250 to 700 nm was used.

### Determination of the mitochondrial activity

The mitochondrial function of the incubated cells was analyzed using the WST-1 assay (Roche Diagnostics GmbH). This assay is based on the cleavage of stable tetrazolium salt WST-1 by metabolically active cells to an orange formazan dye. The WST-1 assay was performed according to the manufacturer's instructions, with appropriate controls. After nanoparticle exposure, the cells were incubated with the ready-to-use WST-1 reagent for 4 h. After this incubation period, formazan formation was quantified by absorbance measurements at 650 nm. The net absorbance change taken from the wells of untreated cultured cells was scaled to 100% cell viability.

### Determination of cell viability by FDA/PI staining

To differentiate between viable and dead cells, fluorescein diacetate (FDA)/propidium iodide (PI) (Sigma-Aldrich, Deisenhofen, Germany) staining was performed. FDA, a membrane permeable dye, is metabolized by viable cells to a green fluorescent dye. PI intercalates in nucleic acids and is unable to penetrate the cell membrane and therefore stains membrane-damaged cells only. Cell viability of A549 cells was determined by culturing the cells for 48 h on the Si_3_N_4 _membrane at 37°C and 5% CO_2_. Afterwards, the cells were treated with the fluorescent dye mixture for 15 s. Thereafter, the cells were washed once with PBS to remove excessive dye molecules. The cells were observed under a Zeiss Observer Z1 fluorescence microscope (Zeiss, Jena, Germany) using ex_FDA _470 nm/em_FDA _525 nm and ex_FDA _555 nm/em_FDA _602 nm. The percentage of the viable and dead cells was analyzed using the cell imaging analysis software.

### Time-lapse microscopy

Time-dependent nanoparticle-induced GFP expression was quantified using time-lapse microscopy. The system Biostation IM-Q (Nikon, Düsseldorf, Germany) is composed of a microscope, an incubator, and a high-resolution camera. The combination of an LED and a fluorescence filter provides the opportunity for fluorescence quantification. pIL8-GFP A549 cells were exposed to 30 μg/ml GC10 or SC10 in the micro culture chamber at 37°C for 48 h. Every hour, a phase contrast and a fluorescent (ex 472 nm/em 520 nm) image of the entire cell culture area were taken of the same section of the sample. The amount of fluorescent cells was quantified via the software analysis.

### Scanning electron microscopy

For SEM of adipogenic differentiated hMSCs, neuronal differentiated PC-12 cells, and A549 cells, the cells were fixed with cacodylate/glutaraldehyde buffer and contrasted using 2% osmium tetroxide and 1% tannic acid. After dehydrating the cells with ethanol, they were dried in a critical point dryer CPD-7501 (Quorum Technologies Ltd., East Sussex, UK) and covered with gold. Samples were examined in a scanning electron microscope EM 109T (Zeiss) using secondary electron mode.

### Quantification of pIL8-GFP induction

For quantification of an induced inflammatory reaction, the reporter cells pIL8-GFP A549 were used. The induction of the promoter is linked to the production of the inflammatory cytokine, and consequently, the intensity of the IL8 promoter activation is proportional to the GFP expression, which could be quantified microscopically and fluorometrically. For fluorometric quantification of GFP expression 300 to 10,000 cells per well were exposed to GC10 or SC10 (0 to 50 μg/ml) for 24 h at 37°C. After washing the cells with PBS, the GFP intensity (ex 485 nm/em 535 nm) was quantified fluorometrically using a Tecan plate reader (Tecan Deutschland GmbH, Crailsheim, Germany). RhTNF-alpha, as inflammation stimulating agent, was used as positive control. The IL8 promoter activation of pIL8-GFP A549 cells cultured in the micro cell culture chambers was analyzed after SC10 or GC10 exposure (24 h) by fluorescence microscopy.

### Statistical analysis

Independent experiments were performed three times in triplicates (*n *= 9), and the data are presented as mean ± SD. Statistical significance was established as *p *< 0.01. Statistical tests were performed using the Mann-Whitney *U *test.

## Abbreviations

Bio-MEM: biological microelectromechanical systems; DNA: deoxyribonucleic acid; FCS: fetal calf serum; GC: gold colloidal nanoparticles; GFP: green fluorescent protein; hMSCs: human mesenchymal stem cells; MC: magnetite colloidal nanoparticles; MCC: microcavity chip; OECD: Organisation for Economic Co-operation and Development; PBS: phosphate-buffered saline; PCR: polymerase chain reaction; pIL8: interleukin-8 promoter; PMMA: polymethylmethacrylate; REACH: Registration: Evaluation: Authorisation and Restriction of Chemicals; rhTNF-alpha: recombinant tumor necrosis factor-alpha; SC: silver colloidal nanoparticles; Si_3_N_4_: silicon nitride; TNF: tumor necrosis factor.

## Competing interests

The authors declare that they have no competing interests.

## Authors' contributions

YK carried out the cytotoxicity studies, performed the nanoparticle characterization, designed the miniaturized cell culture chambers, participated in their fabrication, and wrote the main parts of the manuscript. GO carried out the transfection of the reporter cell line and was involved in the preparation of the manuscript. AS fabricated the microcavity chip. AD and HT obtained funding for this project and helped to draft the manuscript. HB helped to draft the manuscript. All authors read and approved the final manuscript.

## References

[B1] Arora S Jain J Rajwade JM Paknikar KM Cellular responses induced by silver nanoparticles: in vitro studies Toxicol Lett 2008 179 93 100 10.1016/j.toxlet.2008.04.009 18508209

[B2] Choi SJ Oh JM Choy JH Toxicological effects of inorganic nanoparticles on human lung cancer A549 cells J Inorg Biochem 2009 103 463 471 10.1016/j.jinorgbio.2008.12.017 19181388

[B3] Lanone S Rogerieux F Geys J Dupont A Maillot-Marechal E Boczkowski J Lacroix G Hoet P Comparative toxicity of 24 manufactured nanoparticles in human alveolar epithelial and macrophage cell lines Part Fibre Toxicol 2009 6 14 10.1186/1743-8977-6-14 19405955 PMC2685765

[B4] Lewinski N Colvin V Drezek R Cytotoxicity of nanoparticles Small 2008 4 26 49 10.1002/smll.200700595 18165959

[B5] Uboldi C Bonacchi D Lorenzi G Hermanns MI Pohl C Baldi G Unger RE Kirkpatrick CJ Gold nanoparticles induce cytotoxicity in the alveolar type-II cell lines A549 and NCIH441 Part Fibre Toxicol 2009 6 18 10.1186/1743-8977-6-18 19545423 PMC2705341

[B6] Ying E Hwang HM In vitro evaluation of the cytotoxicity of iron oxide nanoparticles with different coatings and different sizes in A3 human T lymphocytes Sci Total Environ 2010 408 4475 4481 10.1016/j.scitotenv.2010.07.025 20673962

[B7] Anderson EC Hessman C Levin TG Monroe MM Wong MH The role of colorectal cancer stem cells in metastatic disease and therapeutic response Cancers (Basel) 3 319 339 10.3390/cancers3010319 PMC3036171 21318087

[B8] Rajaraman R Guernsey DL Rajaraman MM Rajaraman SR Stem cells, senescence, neosis and self-renewal in cancer Cancer Cell Int 2006 6 25 10.1186/1475-2867-6-25 17092342 PMC1664585

[B9] Fitzpatrick LA Leong DA Individual parathyroid cells are more sensitive to calcium than a parathyroid cell population Endocrinology 1990 126 1720 1727 10.1210/endo-126-3-1720 2407521

[B10] Petrasek D Samtaney R Cohen DS Glandular regulation of interstitial diffusion: a model and simulation of a novel physiological mechanism Am J Physiol Endocrinol Metab 2002 283 E195 206 12110523 10.1152/ajpendo.00306.2001

[B11] Sun F Maercklein P Fitzpatrick LA Paracrine interactions among parathyroid cells: effect of cell density on cell secretion J Bone Miner Res 1994 9 971 976 7942165 10.1002/jbmr.5650090703

[B12] Arkhipov SN Berezovski M Jitkova J Krylov SN Chemical cytometry for monitoring metabolism of a Ras-mimicking substrate in single cells Cytometry A 2005 63 41 47 15584019 10.1002/cyto.a.20100

[B13] Das G La Rocca R Lakshmikanth T Gentile F Tallerico R Zambetti LP Devitt J Candeloro P De Angelis F Carbone E Di Fabrizio E Monitoring human leukocyte antigen class I molecules by micro-Raman spectroscopy at single-cell level J Biomed Opt 2010 15 027007 10.1117/1.3368687 20459281

[B14] De Angelis F Das G Candeloro P Patrini M Galli M Bek A Lazzarino M Maksymov I Liberale C Andreani LC Di Fabrizio E Nanoscale chemical mapping using three-dimensional adiabatic compression of surface plasmon polaritons Nature Nanotechnology 2010 5 67 72 19935647 10.1038/nnano.2009.348

[B15] Ebert A Tittmann BR Du J Scheuchenzuber W Technique for rapid in vitro single-cell elastography Ultrasound Med Biol 2006 32 1687 1702 10.1016/j.ultrasmedbio.2006.06.002 17112955

[B16] Hu S Le Z Krylov S Dovichi NJ Cell cycle-dependent protein fingerprint from a single cancer cell: image cytometry coupled with single-cell capillary sieving electrophoresis Anal Chem 2003 75 3495 3501 10.1021/ac034153r 14570202

[B17] Hu S Le Z Newitt R Aebersold R Kraly JR Jones M Dovichi NJ Identification of proteins in single-cell capillary electrophoresis fingerprints based on comigration with standard proteins Anal Chem 2003 75 3502 3505 10.1021/ac034154j 14570203

[B18] Krylov SN Arriaga E Zhang Z Chan NW Palcic MM Dovichi NJ Single-cell analysis avoids sample processing bias J Chromatogr B Biomed Sci Appl 2000 741 31 35 10.1016/S0378-4347(99)00539-3 10839129

[B19] Lantz AW Bao Y Armstrong DW Single-cell detection: test of microbial contamination using capillary electrophoresis Anal Chem 2007 79 1720 1724 10.1021/ac061770h 17297979

[B20] Roers A Hansmann ML Rajewsky K Kuppers R Single-cell PCR analysis of T helper cells in human lymph node germinal centers Am J Pathol 2000 156 1067 1071 10.1016/S0002-9440(10)64974-7 10702422 PMC1876856

[B21] Yun KS Yoon E Micro/nanofluidic device for single-cell-based assay Biomed Microdevices 2005 7 35 40 10.1007/s10544-005-6169-5 15834518

[B22] Zhang H Buchholz TA Hancock D Spitz MR Wu X Gamma-radiation-induced single cell DNA damage as a measure of susceptibility to lung cancer: a preliminary report Int J Oncol 2000 17 399 404 10891553 10.3892/ijo.17.2.399

[B23] Hofstadler SA Severs JC Smith RD Swanek FD Ewing AG Analysis of single cells with capillary electrophoresis electrospray ionization Fourier transform ion cyclotron resonance mass spectrometry Rapid Commun Mass Spectrom 1996 10 919 922 10.1002/(SICI)1097-0231(19960610)10:8<919::AID-RCM597>3.0.CO;2-8 8777325

[B24] Gotz S Karst U Recent developments in optical detection methods for microchip separations Anal Bioanal Chem 2007 387 183 192 17031620 10.1007/s00216-006-0820-8 PMC7080113

[B25] James CD Reuel N Lee ES Davalos RV Mani SS Carroll-Portillo A Rebeil R Martino A Apblett CA Impedimetric and optical interrogation of single cells in a microfluidic device for real-time viability and chemical response assessment Biosens Bioelectron 2008 23 845 851 10.1016/j.bios.2007.08.022 17933506

[B26] Vandaveer WRt Pasas-Farmer SA Fischer DJ Frankenfeld CN Lunte SM Recent developments in electrochemical detection for microchip capillary electrophoresis Electrophoresis 2004 25 3528 3549 10.1002/elps.200406115 15565707

[B27] Di Carlo D Wu LY Lee LP Dynamic single cell culture array Lab Chip 2006 6 1445 1449 10.1039/b605937f 17066168

[B28] Betty CA Porous silicon: a resourceful material for nanotechnology Recent Pat Nanotechnol 2008 2 128 136 10.2174/187221008784534514 19076047

[B29] Davidsson R Genin F Bengtsson M Laurell T Emneus J Microfluidic biosensing systems. Part I. Development and optimisation of enzymatic chemiluminescent micro-biosensors based on silicon microchips Lab Chip 2004 4 481 487 10.1039/b400894d 15472732

[B30] Davidsson R Johansson B Passoth V Bengtsson M Laurell T Emneus J Microfluidic biosensing systems. Part II. Monitoring the dynamic production of glucose and ethanol from microchip-immobilised yeast cells using enzymatic chemiluminescent micro-biosensors Lab Chip 2004 4 488 494 10.1039/b400900b 15472733

[B31] Fischer R Steinert S Frober U Voges D Stubenrauch M Hofmann GO Witte H Cell cultures in microsystems: biocompatibility aspects Biotechnol Bioeng 2011 108 687 693 10.1002/bit.22951 20872818

[B32] Stangegaard M Petronis S Jorgensen AM Christensen CB Dufva M A biocompatible micro cell culture chamber (microCCC) for the culturing and on-line monitoring of eukaryote cells Lab Chip 2006 6 1045 1051 10.1039/b603379b 16874376

[B33] Uhlemann J Lender M Freyer R Biocompatibility of glass and silicon Biomed Tech (Berl) 1998 43 Suppl 438 440 9859434

[B34] Cho YK Shin H Lee SK Kim T Current application of micro/nano-interfaces to stimulate and analyze cellular responses Ann Biomed Eng 2010 38 2056 2067 10.1007/s10439-010-9984-7 20213211

[B35] Gomez-Sjöberg R Leyrat AA Pirone DM Chen CS Quake SR Versatile, fully automated, microfluidic cell culture system Anal Chem 2007 79 8557 8563 10.1021/ac071311w 17953452

[B36] Kawazoe N Guo L Wozniak MJ Imaizumi Y Tateishi T Zhang X Chen G Adipogenic differentiation of mesenchymal stem cells on micropatterned polyelectrolyte surfaces J Nanosci Nanotechnol 2009 9 230 239 10.1166/jnn.2009.J003 19441301

[B37] Lee PJ Hung PJ Rao VM Lee LP Nanoliter scale microbioreactor array for quantitative cell biology Biotechnology and Bioengineering 2005 94 5 14 10.1002/bit.20745 16315325

[B38] Prokop A Prokop Z Schaffer D Kozlov E Wikswo J Cliffel D Baudenbacher F NanoLiterBioReactor: long-term mammalian cell culture at nanofabricated scale Biomed Microdevices 2004 6 325 339 15548879 10.1023/B:BMMD.0000048564.37800.d6

[B39] Simone G Perozziello G Ca^2+ ^mediates the adhesion of breast cancer cells in self-assembled multifunctional microfluidic chip prepared with carbohydrate beads Micro and Nanosystems 2010 2 261 268

[B40] Zhang Z Perozziello G Boccazzi P Sinskey AJ Geschke O Jensen KF Microbioreactors for bioprocess development JALA 2007 12 143 151

[B41] Jin GZ Kim M Shin US Kim HW Effect of carbon nanotube coating of aligned nanofibrous polymer scaffolds on the neurite outgrowth of PC-12 cells Cell Biol Int 2011 35 741 745 PMID:21332449 10.1042/CBI20100705 21332449

[B42] Waddell RL Marra KG Collins KL Leung JT Doctor JS Using PC12 cells to evaluate poly(caprolactone) and collagenous microcarriers for applications in nerve guide fabrication Biotechnol Prog 2003 19 1767 1774 10.1021/bp034086m 14656154

[B43] Liu D Yi C Zhang D Zhang J Yang M Inhibition of proliferation and differentiation of mesenchymal stem cells by carboxylated carbon nanotubes ACS Nano 2010 4 2185 2195 10.1021/nn901479w 20218664

[B44] Oostingh GJ Casals E Italiani P Colognato R Stritzinger R Ponti J Pfaller T Kohl Y Ooms D Favilli F Leppens H Lucchesi D Rossi F Nelissen I Thielecke H Puntes VF Duschl A Boraschi D Problems and challenges in the development and validation of human cell-based assays to determine nanoparticle-induced immunomodulatory effects Part Fibre Toxicol 2011 8 8 10.1186/1743-8977-8-8 21306632 PMC3045340

[B45] Casals E Pfaller T Duschl A Oostingh GJ Puntes V Time evolution of the nanoparticle protein corona ACS Nano 2010 4 3623 3632 10.1021/nn901372t 20553005

[B46] Diegoli S Manciulea AL Begum S Jones IP Lead JR Preece JA Interaction between manufactured gold nanoparticles and naturally occurring organic macromolecules Sci Total Environ 2008 402 51 61 10.1016/j.scitotenv.2008.04.023 18534664

[B47] Pan Y Neuss S Leifert A Fischler M Wen F Simon U Schmid G Brandau W Jahnen-Dechent W Size-dependent cytotoxicity of gold nanoparticles Small 2007 3 1941 1949 10.1002/smll.200700378 17963284

[B48] Davis RR Lockwood PE Hobbs DT Messer RL Price RJ Lewis JB Wataha JC In vitro biological effects of sodium titanate materials J Biomed Mater Res B Appl Biomater 2007 83 505 511 17471514 10.1002/jbm.b.30823

[B49] Schulze C Schaefer UF Ruge CA Wohlleben W Lehr CM Interaction of metal oxide nanoparticles with lung surfactant protein A Eur J Pharm Biopharm 2010 77 376 383 21056657 10.1016/j.ejpb.2010.10.013

[B50] Poth A Jaeger M Alternative testing - the intelligent way to REACH compliance Proc. 6th World Congress on Alternatives & Animal Use in the Life Sciences 2008 AATEX 14 799 803

[B51] Pittenger MF Mackay AM Beck SC Jaiswal RK Douglas R Mosca JD Moorman MA Simonetti DW Craig S Marshak DR Multilineage potential of adult human mesenchymal stem cells Science 1999 284 143 147 10.1126/science.284.5411.143 10102814

[B52] Frens G Controlled nucleation for the regulation of the particle size in monodisperse gold suspensions Nature Physical Science 1973 241 20 22

